# Supplementary material for: The Importance of Molecular Structure for Textural and Physicochemical Properties of Extruded Wheat Flour
Source: Foods. 2025 May 21;14(10):1829. doi: 10.3390/foods14101829 (PMC12111570; doi:10.3390/foods14101829)
Supplement: Supplementary file 1 [file foods-14-01829-s001.zip › foods-3608925-supplementary.pdf]

Table S1. Extruder five-zone temperature setting table

| **Sample** | Ⅰ /℃ | Ⅱ /℃ | Ⅲ /℃ | Ⅳ /℃ | Ⅴ /℃ |
|---|---|---|---|---|---|
| ECM66-170-100 | 60 | 140 | 150 | 160 | 170 |
| ECM66-170-130 | 60 | 140 | 150 | 160 | 170 |
| ECM66-170-160 | 60 | 140 | 150 | 160 | 170 |
| ECM66-170-190 | 60 | 140 | 150 | 160 | 170 |
| ECM66-150-190 | 60 | 120 | 130 | 140 | 150 |
| ECM66-190-190 | 60 | 160 | 170 | 180 | 190 |
| ECM55-170-190 | 60 | 140 | 150 | 160 | 170 |
| ECM104-170-190 | 60 | 140 | 150 | 160 | 170 |
| EWW66-170-190 | 60 | 140 | 150 | 160 | 170 |

ECM66, ECM55, ECM104, and EWW denote extruded wheat flours.
